# Impact of morphine dependency and detoxification by methadone on male’s rat reproductive system

**Published:** 2015-05

**Authors:** Mahnaz Ghowsi, Namdar Yousofvand

**Affiliations:** 1 *Department of Biology, Faculty of Sciences, Razi University, Kermanshah, Iran.*; 2 *Social Development and Health Promotion Research Center, Kermanshah University of Medical Sciences, Kermanshah, Iran.*

**Keywords:** *Morphine*, *Methadone*, *Detoxification*, *Reproductive system*, *Rat*

## Abstract

**Background:**

One of the problems that addicts suffer from is decreased libido. Erectile dysfunction has been reported in men using opioids for treatment of heroin addiction.

**Objective:**

The study was performed to investigate the effects of morphine and detoxification with methadone as causes of sexual dysfunction in addiction.

**Methods and Methods:**

A total of 40 adult male rats (Wistar) were used. Animals were divided in to 4 groups. Control groups received saline for 30 days. Other 2 groups received 10 mg/kg morphine on day 1 and the morphine doses increased daily by 2 mg/kg increments per day until in day 30 a maximum of 68 mg/kg twice daily was achieved. Withdrawal syndrome sings were evaluated. At the end of period, one group of 2 morphine dependent groups was treated with methadone during 14 days. Animals in group 4 (saline solution+ methadone) received saline for 30 consecutive days and then detoxified with methadone during 14 days. Partial weights of seminal vesicles, testes, prostates, seminal vesicles content, concentrations of luteinizing hormone, follicle stimulating hormone, and testosterone in serum were determined.

**Results:**

In the dependent group serum levels of testosterone (p<0.001), folicle stimulating hormone (p=0.0097) and luteinizing hormone (p=0.0031) as well as the weights of testes (p=0.0051), partial weights of prostates, seminal vesicles and seminal vesicles contents (p<0.001) were reduced as compared with control group. In the morphine dependent animals detoxified with methadone, testosterone concentrations and seminal vesicles contents remained lower than levels in the control group (p<0.001).

**Conclusion:**

The results suggest that morphine dependence may impair the reproductive function in male rats.

## Introduction

Illicit substance abuse is a widespread problem among the various populations throughout the world and refers to excessive or repeated use of drug in a way that is damaging to self, community, or both (1). Therefore research on addiction is very important. In Iran opium is one of the most prevailing drugs among addicted subjects (2). Opioid dependent patients have 3 major ways available to manage their addiction: opioid detoxification, agonist maintenance, and antagonist maintenance. Opioid detoxification causes both preventing acute withdrawal and keeping long-term avoidance (3). Three chief treatment modalities used for detoxification during the first medical care of opioid dependent patients are: those using opioid agonists, those using non opioid medications, and the newest modalities of rapid and ultra-rapid opioid detoxification (3). For opioid-based detoxification, methadone, a long-acting µ-opioid receptor agonist, is frequently used agent (3). One of the problems that addicts suffer from is decreased libido. Erectile dysfunction has been reported in men using opoioids for treatment of heroin addiction (4, 5).

It has been reported that consuming oral opioids such as methadone were associated with hypogonadism in 89% of men and also resulted in decreased levels of estradiol, dihydrotestosterone, luteinizing hormone (LH), and follicle stimulating hormone (FSH) (6). These defects may be due to the adverse effect of addictive drugs on gonads and hormones that in turn affects libido and reproduction. It has found that exposure to morphine, the major alkaloid of opium, or opioid peptides caused disorders in the neuroendocrine regulation of release of hormones from anterior pituitary and particularly depressed LH secretion (7). Functional disorder of hypotalamo- pituitary- gonadal (HPG) axis will lead to improper spermatogenesis, reduction of sperm count, semen quality, impairment of erection and lastly infertility (8).

Studies indicate that morphine has an inhibitory influence on gonadotropin releasing hormone (GnRH) (9). Because of effects of opioids on sexual hormones changes in levels of these hormones can affect sperm quality and quantity in male fertility effects of chronic use of opium on sexual hormones may lead to reproductive dysfunction (10). Despite many researches on the effects of morphine on different body systems, the effects of long term opium consumption and detoxification with methadone on endocrine system especially on sex hormones are not well studied.

Therefore, this study investigated the effects of morphine and its detoxification with methadone on sexual function of male rats.

## Materials and methods

**Animals**

A total of 40 adult male Wistar rats were housed in groups of four per cage in animal room, in Razi University and subjected to a 2- week acclimatization period. The animals were maintained under controlled conditions (23±1^o^C, 12hr light/dark cycle, relative humidity of 30-40%) and had access to a rodent laboratory diet (Gharbdaneh, Kermanshah) and drinking tap water.

**Experimental design** **and setting**

The study protocol was approved by ethical committee of Biology Department of Razi University. Morphine sulfate was subcutaneously injected twice a day, for 30 consecutive days. In order to evaluate behavioral signs of withdrawal syndrome in two groups, naloxone (NX) was injected 4 mg/kg intra-peritoneally. Morphine sulfate (TEMAD-Iran) was dissolved in normal saline solution (dissolved at 40^o^C) and was injected subcutaneously. Naloxone (Darupakhsh, Iran) was injected intra-peritoneally.

Methadone syrup (Darupakhsh, Iran) dissolved in the tap water consumed by animals. Rats were randomly divided into four groups, each comprising 8-12 rats as follows:

Group 1 (control, n=12) received normal saline solution for 30 consecutive days.

Group 2 (morphine, n=12) received variable doses of morphine sulfate solution for 30 consecutive days. In first day, 10 mg/kg morphine was injected subcutaneously twice daily with the injections 9 hr apart. The morphine dose was increased daily by 2 mg/kg increments per day until a maximum of 68 mg/kg twice daily was achieved.

Withdrawal syndrome in morphine-dependent group (group 2) and the control group were evaluated by injection of 4 mg/kg naloxone in four animals from each group and then eliminated from related groups. NX (4 mg/kg) was given 1 hr after the last morphine injection in morphine-dependent groups. Significant behavioral signs of withdrawal syndrome such as jumping, exploring, wet dog shakes, escape attempts, and irritability to handling were investigated. These two subgroups used for withdrawal test only.

Group 3 of rats (morphine+methadone, n=8) received variable doses of morphine sulfate solution for 30 consecutive days. In first day, 10 mg/kg morphine was injected subcutaneously twice daily with the injections 9 hr apart. The morphine dose was increased daily by 2 mg/kg increments per day until a maximum of 68 mg/kg twice daily was achieved and then detoxified with methadone for 14 days.

Animals in group 4 (saline solution+ methadone, n=8) received saline for 30 consecutive days and then detoxified with methadone during 14 days.

**Detoxification and estimation of methadone consumption**

Detoxification of opioid dependence in animals was performed according to the protocol offered by the Ministry of Health and Medical Education (11) for human model. Period of detoxification was 14 days. Doses of methadone syrup after being dissolved in the tap water were poured in a container of water and were accessible to the animals. Changes in the methadone concentrations were according to the method of detoxification in which dose of methadone is 10-30 mg in the first 24 hr and maximum dose should not exceed 60 mg in 24 hr (11). The amount of water consumed by animals was also determined (Table I). The mean amount of drinking water per 24 hr (40±2 ml) for each rat was used to determine daily methadone intake and its concentration in drinking water (Table I).

Concentration of methadone in water was 1mg/100ml and daily methadone intake by each rat in 24 hr was 0.4±0.02 mg on day 1 and the methadone concentrations increased daily until concentration of methadone in water was 2.5 mg/100ml in day 7 a maximum of 1±0.05 mg daily was achieved. Then the methadone concentrations decreased daily until in day 14 a minimum of 0.4±0.02 mg daily was achieved.

**Hormone analysis**

At the end of all procedures mentioned above, animals were anesthetized with chloroform and blood samples were collected by cardiac puncture. After clotting, blood samples were centrifuged at 1,500 g or rpm for 15 min. The serum specimens were collected from these samples were frozen at -70^o^C. After collection of all specimens, serum levels of LH and FSH were measured by immunoradiometry assay (IRMA) technique. The serum levels of LH and FSH were determined, using the RADIM (Rom-Italy) kits. For FSH kit the intra-assay coefﬁcient of variation was 6.2% and the interassay coefﬁcient of variation was 6.5% and the sensitivity of the assay was 0.18mIU/ml. For LH kit the intraassay coefficient of variation was 7.8% and the interassay coefficient of variation was 8.2% and the sensitivity of the assay was 0.2mIU/ml. The serum concentrations of total testosterone were measured by Radioimmunoassay (RIA) technique using the Bio Source Testo- RIA- CT Kits. The intra-assay coefficient of variation was 4%, and the interassay coefficient of variation was 8%. The sensitivity of the assay was 0.05ng/ml.

**Body weights differences, testis weight and the ratio of testicles weights to the body weight**

Each rat was weighed. The animals were then anaesthetized using chloroform, and their testicles, prostates and seminal vesicles were removed surgically, washed and weighed. The ratios of testicles, prostate, and seminal vesicles weights to the body weight (Partial weights of these organs) were calculated and showed as percentage (12).

**Statistical analysis**

Data are shown as mean±SEM of the variables. For statistical analysis one-way analysis of variance (ANOVA) followed by Tukey’s post-hoc test was used. Differences with p<0.05 were considered significant. To calculate the exact amount of p-value, data were compared between the each two groups by the Student’s *t*-test.

## Results

**Behavioral signs of withdrawal syndrome**

After naloxone administration, significant behavioral changes such as jumping, exploring, wet dog shakes, escape attempts, and irritability to handling were observed in morphine administered rats but this signs did not observed in control group (13). Thus, morphine dependence and withdrawal syndrome models were established in rats. But, due to the aim of this study and the shortness of time, we did not count these behavioral signs.

**Serum levels of testosterone, LH and FSH**

To evaluate effects on reproductive system induced by the morphine and detoxification with methadone, serum concentrations of some important sex hormones were determined (Table II). The LH levels in the morphine group were significantly lower than the control group (p=00031), but no significant difference were seen in the LH levels between the morphine+methadone group and control group. The testosterone concentrations in the morphine and morphine+methadone groups were reduced as compared to control group (p<0.001). The FSH levels in the morphine group were significantly lower than the FSH levels in the control group (p=0.0097).

**Partial weights of prostates and seminal vesicles and seminal vesicles contents**

Some parameters that indicate effects of morphine and detoxification with methadone on reproductive organs are listed in table III. No significant differences were found between the experimental and the control group for the partial weights of testes. The testes weights in the morphine group were significantly lower than the control group (p=0.0051). As seen in table III, the partial weights of prostates in the morphine group was reduced as compared to the control group (p<0.001). Prostate weights, in the morphine group were significantly lower than the control group (p<0.001). Partial weights of seminal vesicles were significantly decreased in the morphine group in comparison with the control group (p<0.001). Seminal vesicle weights in the morphine group were significantly lower than control group (p<0.001). The seminal vesicle contents in the morphine and morphine+methadone groups were lower than the control group (p<0.001).

**Table I:** Estimate of methadone consumption (intake) via drinking water in two groups of animals

** Parameter**	**Daily water intake by each rat (ml)**	**Duration of methadone exposure (day)**
**Groups**		
Morphine + Methadone	40 ± 2.00	14
Saline+ Methadone	40 ± 1.99	14

**Table II:** Effects of morphine and detoxification with methadone on sex hormones in the heading of each group columns add Mean±SEM

** Groups**	**Control**	**Morphine**	**Morphine + Methadone**	**Saline+ Methadone**
**Parameter**				
FSH (mIu/ml)	0.20 ± 0.01 (n=7)	0.16 ± 0.01 (n=7)	0.18 ± 0.01 (n=7)	0.19 ± 0.01 (n=7)
LH (mIu/ml)	1.49 ± 0.28 (n=7)	0.45 ± 0.02** (n=7)	0.91 ± 0.19 (n=7)	1.10 ± 0.11 (n=7)
Testosterone (ng/ml)	5.04 ± 1.01 (n=6)	0.51 ± 0.24*** (n=6)	0.62 ± 0.46^***^ (n=6)	2.70 ± 0.87 (n=5)

** Significantly different (p=0.0031) than control group (One-way analysis of variance followed by Tukey’s post-hoc test).

*** significantly different (p<0.001) than in the control group (One-way analysis of variance followed by Tukey’s post-hoc test).

**Table III:** Effects of morphine and detoxification with methadone on weights of testes, prostates, and seminal vesicles

** Groups**	**Control** **(n=8)**	**Morphine** **(n=7)**	**Morphine + Methadone** **(n=7)**	**Saline + Methadone ** **(n=8)**
**Organ**				
Testes (mg)	3005± 132.0	2334 ± 151.3^**^	2459 ± 187.2	2814 ± 168.0
Partial weights of Testes (%)	0.987 ± 0.039	0.911 ± 0.04	0.859 ± 0.01	0.982 ± 0.07
Prostate (mg)	602.4 ± 42	251.0 ± 34^***^	452.0 ± 41	631.0 ± 46
Partial weights of Prostates (%)	0.20 ± 0.01	0.10 ±0.01^***^	0.16 ± 0.01	0.22 ± 0.02
Seminal vesicles (mg)	706 ± 56	249 ± 33^***^	546 ± 67	681 ± 84
Partial weights of Seminal vesicles (%)	0.23± 0.02	0.10 ± 0.01^***^	0.19 ± 0.02	0.23 ± 0.02
Seminal vesicles content(ml)	0.52± 0.03	ND ^***^	0.27 ± 0.05^***^	0.41 ± 0.04

The data are expressed as Mean ± SE.

ND: Not determined

*** Significantly different (p<0.001) than in the control group (One-way analysis of variance followed by Tukey’s post-hoc test).

** Significantly different (p=0.0051) than in the control group (One-way analysis of variance followed by Tukey’s post-hoc test).

## Discussion

In this study, the effects of long term opium consumption and detoxification with methadone on reproductive system were evaluated. Reduced libido and erectile dysfunction may reveal that serum testosterone is lower than normal because testosterone performs a considerable role in male sexual function (14). Although sexual dysfunction in patients as an adverse event of opioids was gradually recognized in the literature, most of them had additional diseases and were taking various drugs that may confound the results of endocrine tests (15, 16). 

The ideal situation would have been to use the experimental animals to evaluate the effect of treatment with morphine and detoxification with methadone on reproductive system. Direct evaluation of the drug’s effect on testicular function under in vitro conditions are very useful, but it does not necessarily reflect the actions of these compounds in organism because complete reproducing of the complicated mutual actions between different systems and cells that regulate the status of different hormones under in vivo situations is hard. In the present study, testosterone levels in the Morphine group were lower than the control group. This decrease may be due to a decrease in testis cellular function or may be due to a decrease in release of LH which is consistent with the presumption that changes in testosterone are secondary to changes in LH (17).

Most researchers suggest that reproductive system is affected by opioids at a single place in the HPG axis. Hypothalamus releases luteinizing hormone releasing hormone (LHRH) in turn causes secondary changes in LH and, following testosterone. Some studies have supported this assumption and indicated that the pituitary or gonads were not affected by opioids directly or opioids have small direct effect on them (17, 18). Some evidences by in situ hybridization assessment showed that opioids down-regulate GnRH mRNA levels. These evidences suggest that morphine may decrease the GnRH biosynthesis (19). Moreover, the end-product of the axis, the gonadal sex steroid hormones regulates opioid actions. The feedback inhibition of LH by gonadal steroids is regulated by opioids (10). Some studies in male rats suggested that morphine exposure does not affect LH directly but, it intensifies the responsiveness of the hypothalamus to negative feedback by testosterone (20).

Our results obviously indicate that morphine affected the LH status in the morphine dependent group but since we did not survey LHRH levels, we do not know that this reduction is a subsequent of the direct effect of morphine on the pituitary or that is due to the effect of it on hypothalamus. Some researchers have showed that opioids potently influence gonadal portion of the HPG axis (21-23). Particularly, testes produce endogenous opioid peptides and evidence suggests that testosterone release in testes is inhibited by endogenous opioid peptides (21-26).

According to this evidence, opioid drugs also may directly influence steroidogenesis. In one study the effects of opioids on testicular function were evaluated in the rat by way of assessments of concentration of testosterone in serum, testicular interstitial fluid (TIF) formation and TIF testosterone levels after morphine and opioid antagonist (naloxone, naltrexone) administration. The concentration of testosterone in serum and TIF were significantly reduced 1 to 6 h after morphine (10 mg/kg) injection, and TIF volumes were reduced 2-3 hr after injection morphine. Each of these decreases was dose-related. In contrast to the effects of morphine, the opioid antagonist naloxone increased TIF testosterone but did not alter TIF volumes (27).

The results of present study support the hypothesis that opioids inhibit LH release but direct effects on the testes should be examined. Although detoxification with methadone did not affect significantly the status of the LH in the morphine+methadone group respect to control group, the testosterone levels were yet low. This effect may indicate that chronic morphine exposure inhibits testosterone secretion within the testes directly. No significant differences were seen for the status of the LH and testosterone between the saline+methadone and the control groups.

This effect may indicate that treatment with methadone exert little or no direct effect on the pituitary or gonads, but chronic morphine exposure in animals of the morphine+methadone group influenced testes, and subsequently testosterone secretion decreased. In a study narcotic addicts exposed to heroin for 10 days and detoxified with methadone for 7 days. During methadone withdrawal testosterone concentrations remained reduced but low serum testosterone concentrations recover to base level after methadone detoxiﬁcation (28).

Several studies provided results suggesting that methadone affected sex hormone levels. Willenbring *et al* showed a maximally stimulated level of prolactin in 15 men on methadone maintenance for addiction treatment(mean daily dose of 52.7 mg of methadone, average duration of maintenance 18 months), providing evidence for interference by prolactin as a potential pathway leading to depressed testosterone and, hence, to sexual dysfunction in men on methadone maintenance (29). Cicero *et al* in their 1975 study showed various sexual influences in 29 methadone-maintained male subjects. Ejaculate volume and seminal and prostatic secretions were found to be 50% of those in 43 narcotic-free controls. Serum testosterone levels were, on average, 43% of control subjects’. The mean daily methadone dose in this study population was 67 mg (30).

Cicero replicated comparable results in male rats. Serum levels of LH were not recognizable in rats receiving methadone or morphine and they deduced that methadone may act to decrease levels of testosterone in serum by way of interference with pituitary or hypothalamic regulatory hormones (31). In our study no significant difference were seen in serum levels of LH in the morphine+ methadone group respect to control group. Specifically, our data demonstrate that treatment by morphine influenced FSH levels in animals of morphine dependent group. Data of FSH status in this study are not consistent with a study in which FSH levels were not influenced by opioid administration significantly (29, 32).

Testes weights in the rats of Morphine group were reduced significantly when compared to control group. However, there were no significant differences on the partial weight of testes in experimental and control groups, so this reduction may be due to reduction in the animal weights as a result of drug exposure. Of surveyed parameters partial weights of prostates, partial weights of seminal vesicles and seminal vesicle contents in the morphine dependent group have significant decrease respect to control group.

In the detoxificated animals of the morphine+methadone group partial weights of seminal vesicles restored during detoxification, but, partial weights of prostates and seminal vesicles contents were lower than control group. No significant differences in the status of these parameters were seen between the saline+methadone and control group. These results indicate that morphine exposure affected sex organs so severely that during detoxification its destructive effects didn’t recover. In the male reproductive system prostate and seminal vesicles secretions depend on androgens and lack of androgens induces fast apoptosis in epithelial cells and collapse in the parenchyma of these organs (33).

These effects lead to slow stromal atrophy in these organs. However, testosterone substitution restores initial architectures of prostate and seminal vesicles (34). In our study changes in partial weights of prostates and seminal vesicles and seminal vesicles secretion may be related to altered testosterone status during morphine exposure period. Our observations are similar to the observations of one study that showed a 3-day period of morphine pellet implantation affected the prostates and seminal vesicles and reduced their secretory activity and both the wet and dry tissue weight of the seminal vesicles were decreased (35).

In a study Teusch *et al* reported decline in libido and orgasm dysfunction in men maintained on methadone is more frequently than controls (5). Whether morphine or methadone have direct effects on the secretory cells of reproductive system or not should be further investigated.

## Conclusion

These data and similar studies suggest that morphine abuse may impair the reproductive function in male rat by peripheral and central mechanisms which in turn interfere with normaltestosterone production in testes and may cause decrease in libido and erectile dysfunction. According to the findings of this study, detoxification with methadone had no effect on LH release in the HPG axis, but the testosterone levels were yet low in detoxified rats.

## Note

***This article extracted from M.Sc. thesis (Mahnaz Ghowsi)***
